# Development of a high throughput yeast-based screening assay for human carbonic anhydrase isozyme II inhibitors

**DOI:** 10.1186/s13568-018-0653-9

**Published:** 2018-08-04

**Authors:** Anyaporn Sangkaew, Jerapan Krungkrai, Chulee Yompakdee

**Affiliations:** 1 Department of Microbiology, Faculty of Science, Chulalongkorn University, Bangkok, Thailand; 2 Department of Biochemistry, Faculty of Medicine, Chulalongkorn University, Bangkok, Thailand

**Keywords:** Human carbonic anhydrase isozyme II, *Saccharomyces cerevisiae*, Yeast-based assay, Resazurin, *NCE103*, Carbonic anhydrase inhibitor

## Abstract

Carbonic anhydrase (CA; EC 4.2.1.1) catalyzes the reversible hydration of carbon dioxide (CO_2_) to bicarbonate and proton. There are 16 known isozymes of α-CA in humans, which differ widely in their kinetics, subcellular localization and tissue-specific distribution. Several disorders are associated with abnormal levels of CA, and so the inhibition of CA has pharmacological application in the treatment of many diseases. Currently, searching for novel CA inhibitors (CAI) has been performed using in vitro methods, and so their toxicity remains unknown at the time of screening. To obtain potentially safer CAIs, a screening procedure using an in vivo assay seems to have more advantages. Here, we developed a yeast-based in vivo assay for the detection of inhibitors of the human CA isozyme II (hCAII). The yeast *Saccharomyces cerevisiae* mutant deprived of its own CA (Δ*nce103* strain) can grow under a high CO_2_ condition (5% (v/v) CO_2_) but not at an ambient level. We constructed Δ*nce103* strains expressing various levels of hCAII from a plasmid harboring the hCAII gene arranged under the control of variously modified *GAL1* promoter and relying on the expression of hCAII for growth under low CO_2_ condition. Using a multidrug-sensitive derivative of the Δ*nce103* strain expressing a low level of hCAII, we finally established a high throughput in vivo assay for hCAII inhibitors under a low CO_2_ condition. Cytotoxicity of the candidates obtained could be simultaneously determined under a high CO_2_ condition. However, their inhibitory activities against other CA isozymes remains to be established by further investigation.

## Introduction

Carbonic anhydrases (CAs, EC 4.2.1.1) belong to the metalloenzymes family of proteins. They are a class of enzymes catalyzing the simple but physiologically essential process of carbon dioxide (CO_2_) hydration to bicarbonate and proton (CO_2_ + H_2_O ↔ HCO_3_^−^ + H^+^). Overall, CAs play important roles in pH regulation, fluid balance, carboxylation reactions, calcification, bone resorption, tumorigenicity and in other pathological and physiological processes, such as gluconeogenesis, ureagenesis and lipogenesis (Supuran and Scozzafava 2000). Six different genetically distinct CA families have been described to date; namely the α- (present in vertebrates, bacteria, algae and cytoplasm of green plants), β- (predominantly in bacteria and algae), γ- (mainly in archaea and some bacteria), δ- and ζ- (present in marine diatoms) and ƞ-CA (present in parasite) (Del Prete et al. 2014; Lindskog 1997; Supuran 2008). Sixteen α-CA isozymes have been found in humans, which differ widely in their kinetics, subcellular localization, tissue-specific distribution and susceptibility to different inhibitors. However, they all play important physiological roles, as briefly outlined above (Supuran and Scozzafava 2000). Many human CA isozymes are established therapeutic targets for the treatment of a wide range of disorders (Sly and Hu 1995; Supuran 2008; Supuran and Scozzafava 2000). Indeed, antiobesity, antiepilectic, anticancer and antiglaucoma drugs based on CA inhibitors (CAIs) are presently used, and they target various human CA isoforms (Carta and Supuran 2013; Masini et al. 2013; Monti et al. 2013; Scozzafava et al. 2013; Supuran 2008).

Sulfonamide compounds, which are classical CAI, have been used as commercial drugs for the treatment of glaucoma, epilepsy, edema and altitude sickness (Supuran 2008). However, they can inhibit all CA isoforms nonspecifically, diluting the drug effectiveness and causing undesired side effects from the off-target inhibition. Furthermore, a small but significant percentage of patients cannot be treated with sulfonamide-based compounds owing to their sulfa allergy (Lomelino et al. 2016). Thus, safer CAIs are required. In previous studies, CAIs were screened for by in vitro methods using a biochemical strategy, but this approach has several disadvantages. For example, it provides no information about drug uptake into cells, drug stability and, in particular, the cytotoxicity of the compounds (Bilsland et al. 2013). Such disadvantages could be improved by using an in vivo assay.

The yeast *Saccharomyces cerevisiae* has emerged in the last few decades as a powerful organism for the study of many human enzymes. The deep genetic information available on this organism has allowed it to become an increasingly popular model for pharmacological and/or drug discovery studies (Daniel et al. 2005). The Δ*nce103* null mutation leads to the loss of endogenous CA activity and inhibits the growth of the yeast cells under an ambient CO_2_ concentration (Clark et al. 2004) due to the low level of available bicarbonate ions in the absence of CA activity.

Human carbonic anhydrase isozyme II (hCAII) is the most efficient isozyme in CO_2_ hydration and is a highly abundant CA isozyme in cells (Supuran and Scozzafava 2000). Furthermore, hCAII is the only isozyme so far reported in which its overexpression can complement the growth defect of the yeast Δ*nce103* null mutant strain (Clark et al. 2004). Thus, in this study, we aimed to develop a novel yeast-based assay in a 96-well format for the high throughput screening of CAIs against hCAII.

Here, a highly sensitive Δ*nce103* null yeast strain expressing hCAII was constructed and used for screens in a resazurin microtiter plate assay (REMA). The developed yeast-based assay enables high-throughput, live-cell, target-based screening to identify compounds that could inhibit hCAII activity. Hence, it could be a potential tool for accelerating the discovery of non-sulfonamide-based CAIs to be used for the treatment CA-related diseases, such as glaucoma.

## Materials and methods

### Microbial strains and cultivation media

*Escherichia coli* DH5α [F-endA1 hsdR17 (r − K/m − K) supE44 thi-1 λ-recA1 gyrA96 ΔlacU169 (ϕ80lacZΔM15)] (Thermo Fisher Scientific, USA) was used in the construction and transformation of recombinant plasmids. All clonings were performed using *E. coli* DH5α grown at 37 °C in Luria–Bertani medium (Titan Biotech LTD., India) containing 0.1 g L^−1^ ampicillin (T.P. Drug Laboratories, Thailand) (Sambrook et al. 1989). The *S. cerevisiae* strains used in this study are listed in Table 1. Yeast transformants were grown at 30 °C in synthetic dextrose (SD) medium or synthetic raffinose (SR) medium [6.7 g L^−1^ yeast nitrogen base (YNB) without amino acids (Difco Laboratories, USA) and 20 g L^−1^ of either glucose or raffinose (Difco Laboratories, USA), respectively, containing only essential amino acids (Sigma Aldrich, USA)]. Uracil auxotrophic yeast strains were selected by 5-fluoroorotic acid (5-FOA) medium containing 6.7 g L^−1^ YNB with ammonium sulphate and without amino acids, 20 g L^−1^ glucose, 1 g L^−1^ 5-FOA (Zymo Research, USA) and essential amino acids as well as uracil. For the functional complementation, Western blot analysis and two step quantitative reverse transcription-polymerase chain reaction (qRT-PCR) experiments, the yeast transformants were induced for hCAII expression by cultivation in synthetic galactose (SG) medium (6.7 g L^−1^ YNB without amino acids, 10 g L^−1^ raffinose, 20 g L^−1^ galactose; Difco, USA) containing 0.2 g L^−1^ adenine, 1 g L^−1^ leucine and 0.1 g L^−1^ histidine (SG + Ade + Leu + His). The Δ*nce103* strain was grown under the high-CO_2_ condition (5% (v/v) CO_2_) using an AnaeroPack (Mitsubishi Gas Chemical, Japan).

**Table 1:** Yeast strains used in this study

Yeast strain	Genotype	Reference
W303-1A	*MATa ade2*-*1 his3*-*11 leu2*-*3,112 trp1*-*1 ura3*-*1 can1*-*100*	Yeast Genetic Resources Center, Japan
AS01	*MATa ade2*-*1 his3*-*11 leu2*-*3,112 trp1*-*1 ura3*-*1 can1*-*100 nce103::*loxP	This study
AS02	*MATa ade2*-*1 his3*-*11 leu2*-*3,112 trp1*-*1 ura3*-*1 can1*-*100 nce103::*loxP *erg3::*loxP	This study
BY25929	*MATa ade2*-*1 his3*-*11 leu2*-*3,112 trp1*-*1 ura3*-*1 can1*-*100 yrs1::HIS3 yrr1::TRP1 pdr1::hisG pdr3::hisG*	Yeast Genetic Resources Center, Japan
BY25929.1	*MATa ade2*-*1 his3*-*11 leu2*-*3,112 trp1*-*1 ura3*-*1 can1*-*100 yrs1::HIS3 yrr1::TRP1 pdr1::hisG pdr3::hisG erg3::*loxP-*URA3*-loxP	This study
BY25929.2	*MATa ade2*-*1 his3*-*11 leu2*-*3,112 trp1*-*1 ura3*-*1 can1*-*100 yrs1::HIS3 yrr1::TRP1 pdr1::hisG pdr3::hisG erg3::* loxP	This study
BY25929.3	*MATa ade2*-*1 his3*-*11 leu2*-*3,112 trp1*-*1 ura3*-*1 can1*-*100 yrs1::HIS3 yrr1::trp1::* loxP -*URA3*- loxP *pdr1::hisG pdr3::hisG erg3::* loxP	This study
BY25929.4	*MATa ade2*-*1 his3*-*11 leu2*-*3,112 trp1*-*1 ura3*-*1 can1*-*100 yrs1::HIS3 yrr1::trp1::* loxP *pdr1::hisG pdr3::hisG erg3::* loxP	This study
AS03	*MATa ade2*-*1 his3*-*11 leu2*-*3,112 trp1*-*1 ura3*-*1 can1*-*100 yrs1::HIS3 yrr1::trp1::* loxP *pdr1::hisG pdr3::hisG erg3::*loxP *nce103::*loxP-*URA3*- loxP	This study

### Chemicals

Acetazolamide (AZA) and FK506 were purchased from Sigma Aldrich, USA. Avicennin was a gift from Dr. Warinthorn Chavasiri, Department of Chemistry, Faculty of Science, Chulalongkorn University, Thailand.

### Plasmid construction

All plasmids used in this study are shown in Table 2. The hCAII cDNA was obtained from Krungkrai et al. (2005). A full-length *GAL1* promoter (nucleotide positions 1–451) fused with the Flag epitope tag at the C-terminal of *hCAII* (c-Flag *hCAII*) was obtained by PCR amplification from the pAG414GAL (c-Flag *hCAII*) vector (Panthan 2011) using the oligonucleotide primers pGAL1.4_hCAII Fw and NotI_hCAII Rv (Table 3). Each PCR reaction was performed in a total volume of 50 µL according to the manufacturer’s protocol of KOD-Plus-Neo (Toyobo, Japan) containing 5 µL of 10× buffer for KOD-Plus-Neo, 5 µL of 2 mM dNTP, 3 µL of 25 mM MgSO_4_, 0.75–1.5 µL of 10 µM of each primer, 1 µL of KOD-Plus- Neo and 1–2 µL of DNA template. The reaction was performed for 35 cycles of 95 °C for 30 s, 62 °C for 30 s and 72 °C for 90 s. Purified PCR products were ligated into pGEM-T easy (Promega, USA) and then subcloned via a unique *Not*I site into pRS414 (Addgene, USA), a CEN4-ARS1 plasmid with *TRP1* selection marker, to yield the designated pGAL1.4_hCAII plasmid.

**Table 2:** Plasmids used in this study

Plasmid	Description	Reference
pAG414GAL (C Flag hCAII)	*Amp*^*R*^*, TRP1, CEN, hCAII_*Flag	Panthan (2011)
pRS414	*Amp* ^*R*^ *, URA3, CEN*	Addgene, USA
pGAL1.1_hCAII	*Amp*^*R*^*, URA3, CEN*, *P*_*GAL1.1*_*, hCAII_*Flag	This study
pGAL1.2_hCAII	*Amp*^*R*^*, URA3, CEN*, *P*_*GAL1.2*_*, hCAII_*Flag	This study
pGAL1.3_hCAII	*Amp*^*R*^*, URA3, CEN*, *P*_*GAL1.3*_*, hCAII_*Flag	This study
pGAL1.4_hCAII	*Amp*^*R*^*, URA3, CEN*, *P*_*GAL1.4*_*, hCAII_*Flag	This study
pUG72	*Amp*^*R*^*, loxP*-*URA3*-*loxP*	Euroscarf, Germany

**Table 3:** Oligonucleotide primers used in this study

Primer name	Primer sequence (5′–3′)
A. Primers used in the PCR amplified disruption cassettes for gene disruption	
D-*ERG3*_Fw	ATTTCTATCTTTCTTATCAATTCGTTTTTTCATTCACTTGTCAGCTGAAGCTTCGTACGC
D-*ERG3*_Rv	TCTTGAACGTGAAAGAAAGAAAAAAGATGAGACAAACAAGATAGGCCACTAGTGGATCTG
D-*NCE103*_Fw	TACAAATTTCAATTATTACACATCAGACAGCTGAAGCTTCGTACGC
D-*NCE103*_Rv	CCCCGTCTACTTTGTAAATGTCTTTCTATTTCAATGAATGGTAGGCCACTAGTGGATCTG
D-*TRP1_*Fw	GTCTGTTATTAATTTCACAGGTAGTTCTGGTCCATTGGTGACAGCTGAAGCTTCGTACGC
D-*TRP1_*Rv	CTATTTCTTAGCATTTTTGACGAAATTTGCTATTTTGTGCATAGGCCACTAGTGGATCTG
B. Primers used for the confirmation of successful gene disruption in yeast chromosome	
C-*ERG3*_Fw1	CGAAACGACGCCTTTTGTTGCGATTGTCG
C-*ERG3*_Rv1	ATTTGTGTGCTTCTCTTGACGTTCGTTCG
C-*ERG3*_Fw2	TTCAACAAGTTTCAATAGCTCATCAGTCG
C-*ERG3*_Rv2	GAAATCTTGGGCATTTTAAAGCTTCCAGC
C-*NCE103*_Fw1	GTCACCATGACGCTTATCAAGCC
C-*NCE103*_Rv1	ATCGGGCGTTTACCGTATCGC
C-*NCE103*_Fw2	CTACACCTGGGGTCATGATTAGCC
C-*NCE103*_Rv2	GACATTTGCTGGATCACAGACCG
C. Primers used in the PCR amplification of the *GAL1* promoter derivative fused with *hCAII*	
pGAL1.4 Fw	TACAGCTAAGACTACAAAACGGATTAGAAGCCGCCG
pGAL1.3 Fw	TACAGCTAAGACTACAAACCGAGCGGGCGACAGCCC
pGAL1.2 Fw	TACAGCTAAGACTACAAACCGACGGAAGACTCTCCTC
pGAL1.1 Fw	TACAGCTAAGACTACAAAGCAGATGTGCCTCGCGCC
NotI_hCAII Rv	TTTTCCTTTTGCGGCCGCTTTTTTCCTTTTATTTATCATCATCATCTTTG

To modulate the transcription level of hCAII under the control of the *GAL1* promoter, modification of the number of Gal4 binding sites (Cottier et al. 2006; Giniger et al. 1985; Hong et al. 2008; Liang et al. 1996; Marmorstein et al. 1992) was performed by PCR amplification from the pAG414GAL (c-Flag *hCAII*) plasmid using the desired forward primer (one of pGAL1.3_hCAII Fw, pGAL1.2_hCAII Fw or pGAL1.1_hCAII Fw) with the reverse primer NotI_hCAII Rv (Table 3) to amplify a *GAL1* promoter with either three, two or one Gal4 binding site domains, respectively. All PCR reactions were performed as described above. The resulting plasmids with three, two and one Gal4 binding sites were designated as pGAL1.3_hCAII, pGAL1.2_hCAII and pGAL1.1_hCAII, respectively.

### Gene disruption

A PCR-based gene disruption method (Gueldener et al. 2002) was employed in the yeast strain W303-1A and BY25929 background (Table 1). A disruption cassette, containing the loxP-*URA3*-loxP sequence of pUG72 (EUROSCARF, Germany) flanked at both sides with a short homology sequence of 40 bp at the 5′ and 3′ termini of the target gene, was amplified by PCR using oligonucleotide primers (Table 3) as follows. The disruption cassette loxP-*URA3*-loxP for the *ERG3* gene (Accession No. M64989.1) was amplified using oligonucleotide primers D-*ERG3*_Fw and D-*ERG3*_Rv, whereas the other disruption cassettes were amplified using the primer pairs D-*TRP1_*Fw and D-*TRP1*_Rv for *TRP1* (Accession No. NM_001180315.3) and D-*NCE103_*Fw and D-*NCE103*_Rv for *NCE103* (Accession No. NM_001182875.3).

The respective amplified disruption cassette was transformed into the yeast cells and successful disruptants were confirmed by PCR using the gene-specific oligonucleotide primers (Table 3).

### Yeast transformation

Yeast cells were transformed by the lithium acetate method with 1 µg plasmid or 5 µg DNA fragment, respectively (Gietz et al. 1995). In addition, 50 µg of carrier DNA, deoxyribonucleic acid sodium salt from salmon testes (Sigma Aldrich, USA), was added to enhance the transformation efficiency (Gietz et al. 1995).

### Yeast complementation experiment

Complementation of the ∆*nce103* yeast strain was performed as follows. The exponential growth phase of the yeast transformants in 3 mL SR + Ade + Leu + His medium was diluted to a final 10^6^–10^3^ cells mL^−1^ and 5 µL of each serially diluted culture were spotted onto the surface of SG + Ade + Leu + His agar medium. The plates were incubated at 30 °C for 3–4 days under either the high- or the low-CO_2_ condition.

### Two step quantitative reverse transcription-polymerase chain reaction (qRT-PCR)

The recombinant strains were cultured in SG + Ade + Leu + His medium and then total RNA was extracted using the Masterpure yeast RNA purification kit (Epicentre, USA), as recommended by the manufacturer. For the first stage RT-PCR, aliquots of total RNA (1 µg) were converted into complementary DNA (cDNA) using RevertAid Reverse Transcriptase (Thermo Fisher Scientific, USA) and the respective gene-specific primers (Table 3). All second stage qRT-PCR were run using the SsoAdvanced Universal SYBR Green Supermix (Bio-Rad, USA). The plates containing the qRT-PCR mix were transferred to the CFX Connect Real-Time PCR Detection System (Bio-Rad, USA) and thermal cycled at 95 °C for 3 min followed by 35 cycles of 95 °C for 30 s, 50 °C for 30 s and 72 °C for 30 s. Relative gene expression levels were calculated using the 2^−∆∆CT^ method (Livak and Schmittgen 2001).

### Western blotting analysis

Yeast cells cultured in the inducible (SG + Ade + Leu + His) medium were lysed by vortexing with acid-washed glass beads of 0.45–0.55 diameter (Sigma Aldrich, USA) in sodium dodecyl sulfate (SDS)-sample buffer and subjected to 12% SDS–polyacrylamide gel electrophoresis (SDS-PAGE) and Western blotting analysis using the rabbit anti-Flag (DYKDDDDK tag) primary antibody (Cell Signaling, USA) and horseradish peroxidase conjugated donkey anti-rabbit IgG as the secondary antibody (Cell Signaling, USA). Protein signals were detected by Pierce™ ECL Western Blotting Substrate (Thermo Fisher Scientific, USA).

### Resazurin microtiter plate assay

The REMA was performed in 96-well plates as follows. Briefly, 1 µL of test compound was dissolved in 80 µL of SR + Ade + Leu + His medium to the desired concentration and aliquoted into each well. The AS03(pGAL1.1_hCAII) yeast cells cultivated in SR + Ade + Leu + His medium were then added into each well of the 96-well plate at the appropriate cell density for 10 µL. The test plate was incubated at room temperature for 30 min and then 10 µL of 20% (w/v) galactose was added into each well and incubated at 30 °C under an ambient atmosphere (low CO_2_) or 5% (v/v) CO_2_ condition (high CO_2_) for the appropriate incubation time. A stock solution of 0.1 mg mL^−1^ resazurin sodium salt (Sigma Aldrich, USA) prepared in distilled water was added to each well to a final concentration of 0.03 mg mL^−1^ and further incubated at 30 °C in the dark until the color of the wells without the test compound changed from blue to pink, which indicated the growth of yeast cells. The minimal effective dose was defined as the lowest concentration of the drug that could inhibit the growth of yeast cells and so prevent the color change of resazurin. In addition to the color observation in the test wells, the ratio of the optical densities between resorufin (OD_572_) and resazurin (OD_600_) was evaluated to determine the level of reduction of resazurin to resorufin. Measurement of the yeast culture turbidity at 660 nm (OD_660_) (Amberg et al. 2005) was also performed before the addition of resazurin.

### Statistical analysis

Statistical analysis was performed using the GraphPad Prism 5.01 package (GraphPad Software INC., USA) with one-way analysis of variance, followed by the Dunnett post-test. Each determination was performed in triplicate. Statistical significance was accepted at the p < 0.05 level.

## Results

### Construction of a drug-sensitive derivative of the *nce103* null mutant of *S. cerevisiae* expressing various levels of hCAII from modified *GAL1*-promoter-based expression cassettes

The yeast *S. cerevisiae* is generally highly tolerant to various drugs, which poses a serious obstacle for their use in a yeast-based in vivo drug assay.The yeast strain BY25929 (obtained from the Yeast Genetic Resources Center, Japan; Table 1), was modified to attenuate the general permeability barriers for drugs by disruption of the *ERG3* gene, which is involved in the biosynthesis of ergosterol, a major component of the cell membrane (Hemmi et al. 1995). In addition, the *NCE103* gene, which codes for CA, was also disrupted. The drug-sensitive derivative of the *nce103* null-mutant strain so obtained was designated as the AS03 strain (Table 1).

The AS03 strain was further engineered to express hCAII at various levels. For this purpose, we used the *GAL1* promoter containing four copies of the Gal4-binding site to construct a series of modified promoters containing either four, three, two or one copy(ies) of the Gal4-binding site, as previously described (Cottier et al. 2006; Liang et al. 1996), and these were designated pGAL1.4_hCAII, pGAL1.3_hCAII, pGAL1.2_hCAII or pGAL1.1_hCAII, respectively. The drug-sensitive *nce103* null strain (AS03) was then separately transformed with each of these hCAII expression plasmids.

The expression levels of hCAII in the transformants were compared by multiple methods. The functional complementation of the growth defect by the exogenous hCAII gene was examined by growth of the transformants under the control of *GAL1* promoter under the low-CO_2_ condition. All the transformants harboring the hCAII expression constructs could grow similarly well, while those with the empty vector showed a severe growth defect (data not shown).

**Fig. 1:**
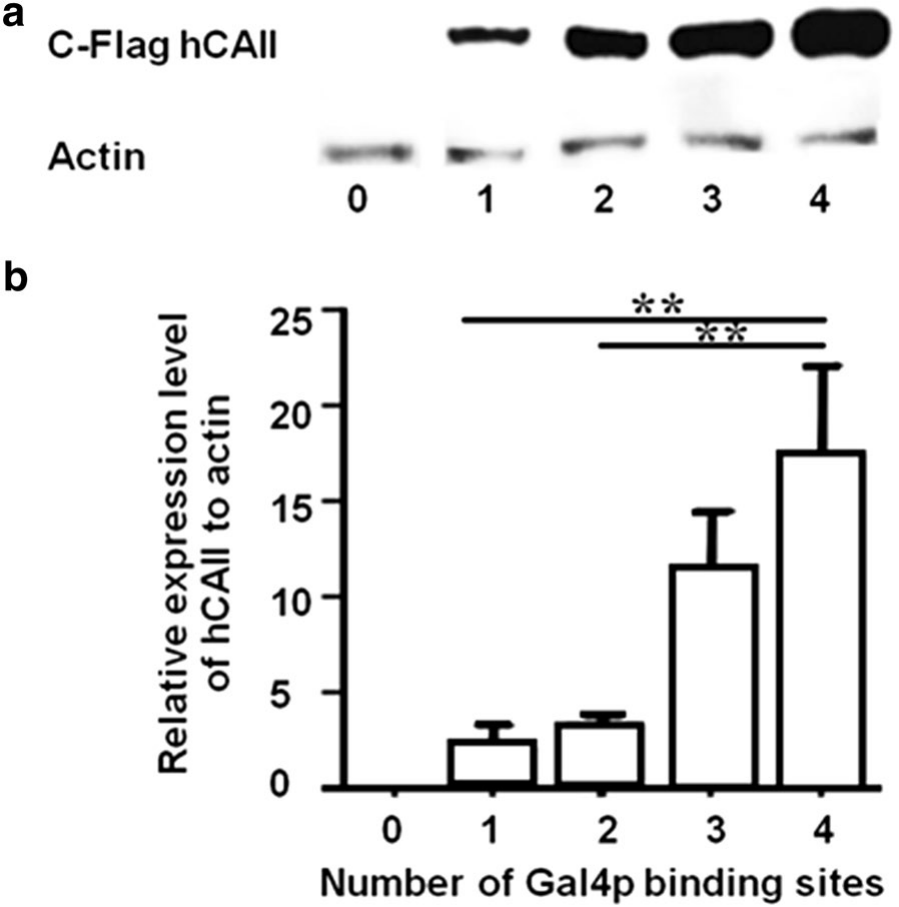
Expression level of hCAII under the control of variously modified *GAL1* promoters. The drug-sensitive AS03 yeast strain harboring either the empty pRS414 vector or one of the *GAL1* promoter derivatives containing either one, two, three or four copies of the Gal4-binding sites (pGAL1.1_hCAII, pGAL1.2_hCAII, pGAL1.3_hCAII or pGAL1.4_hCAII, respectively) was cultivated under a *GAL1*-induced condition for 24 h before harvesting. **a** Protein extracts were prepared and subjected to Western blot analysis using anti-Flag to detect hCAII and anti-β-actin to detect β-actin. Blots shown are representative of those seen from three independent repeats. **b** Total RNA was prepared and converted to cDNA prior to the qRT-PCR analysis of the hCAII mRNA expression level. Data are expressed as the relative level of hCAII mRNA to actin mRNA and shown as the mean expression level, ± 1 SD, and derived from three independent repeats. **Significant difference at p < 0.01 compared to that of the transformant with the pGAL1.4_hCAII plasmid

We next compared the hCAII protein levels of the transformants by Western blot analysis. Protein extracts of the yeast transformants with the plasmid containing an expression construct for the Flag-epitope-tagged hCAII at the C-terminus showed increasing levels of the 29 kDa Flag-hCAII band with the increasing number of the Gal4-binding sites located in the modified *GAL1* promoter of the expression constructs (Fig. 1a). Setting the relative protein levels of the transformants with four Gal4-binding sites as 100%, the levels in the other transformants were 82%, 62% and 39% for three, two and one Gal4-binding sites, respectively.

We further compared the mRNA levels of the transformants by two-step qRT-PCR (Fig. 1b). The obtained hCAII mRNA levels were consistent with the hCAII protein levels.

**Fig. 2:**
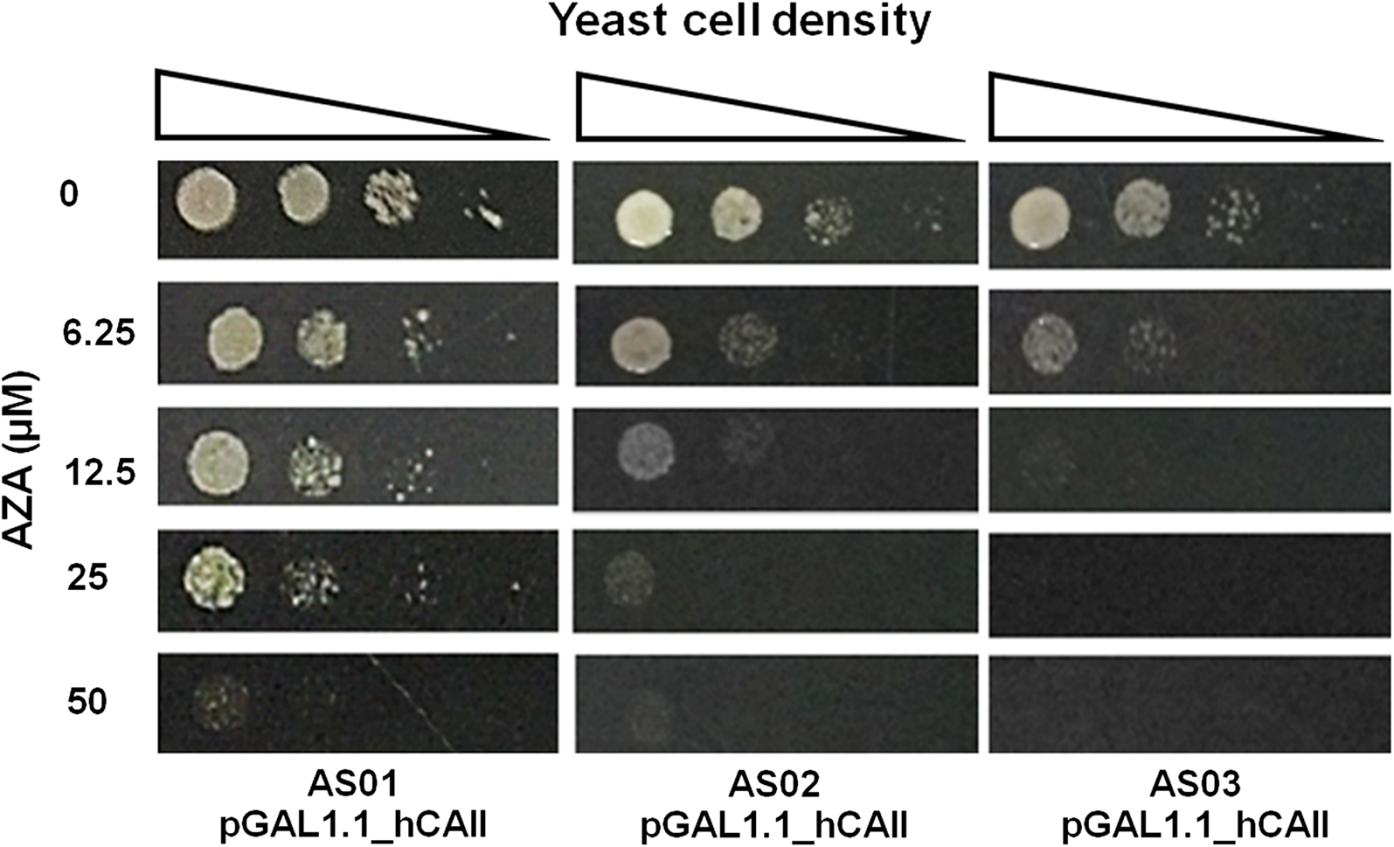
Comparision of the drug-sensitivity phenotype of various *NCE103* null mutant yeast strains. The AS01, AS02 and AS03 cells harboring the pGAL1.1_hCAII plasmid at concentrations of 10^6^, 10^5^, 10^4^ and 10^3^ cells mL^−1^ were spotted onto the surface of SG + Ade + Leu + His plates containing either 0, 6.25, 12.5 or 50 µM AZA and incubated at 30 °C for 3–4 days under a low CO_2_ condition. Images shown are representative from three independent repeats

Next, we compared the sensitivity to AZA, a known CAI including against hCAII (Supuran 2008), of three different yeast strains with deletions of various genes responsible for the general drug resistance of the cells that rely on the exogenous hCAII under the low-CO_2_ condition. Specifically, the strains AS01 (Δ*nce103*), AS02 (Δ*nce103* Δ*erg3*) and AS03 (Δ*nce103* Δ*erg3* Δ*pdr1*Δ*pdr3* Δ*yrr1*) (Table 1) harboring the pGAL1.1_hCAII expression plasmid were constructed and subjected to the drug sensitivity assay on agar plates containing various concentrations of AZA and measuring their growth under the low-CO_2_ condition (spot-test assay). The result revealed that the minimal effective dose of AZA for the transformants of the AS03, AS02 and AS01 strains were 12.5, 25 and 50 µM, respectively (Fig. 2).

To evaluate the most appropriate expression level of hCAII to be used in the indicator cells for the yeast-based assay, the AS03 strain was transformed with the different hCAII expression plasmids and then subjected to the spot test assay on plates containing various concentrations of AZA (0, 3.13, 6.25 and 12.5 μM) under the low-CO_2_ condition. The result showed that the AS03(pGAL1.1_hCAII) transformant which expressed the lowest level of hCAII was the most sensitive strain. It was, therefore, selected for indicator cells in the yeast-based inhibitor assay.

### Setting up a resazurin-yeast-based high throughput screening assay

We next attempted to establish the assay conditions by which hCAII inhibitors could be identified using the AS03(pGAL1.1_hCAII) transformant as the assay cells under the low-CO_2_ condition.

Resazurin is an oxidation–reduction dye that changes color from blue (resazurin) to pink (resorufin) and further to colorless (hydroresorufin) after reduction by living cells (Rampersad 2012). We chose to examine the viability of the indicator cells in liquid culture having resazurin as an indicator dye in a 96 well-plate format.

The AS03(pGAL1.1_hCAII) indicator cells were cultivated in the SG + Ade + Leu + His medium for the induced expression of hCAII under the low-CO_2_ condition in the absence or presence of AZA as a CAI (Figs. 4 and 5).

To optimize the initial density of the assay cells, we compared the effect of the cell density on the color reaction by varying the initial seeding density from 1 to 100 × 10^4^ cells mL^−1^. As shown in Fig. 4, the optimal initial cell densities were found to be between 0.5 and 1 × 10^5^ cells mL^−1^. The OD_572_/OD_600_ ratio in the wells containing AZA was approximately half of that in the negative control well. However, when higher cell densities (between 0.5 and 1 × 10^6^ cells mL^−1^) were used, the OD_572_/OD_600_ ratio in the wells containing AZA was higher than that in the negative control well. In contrast, when a lower cell density (1 × 10^4^ cells mL^−1^) than the optimal one was used, the ratios in the wells with or without AZA did not show any significant difference (Fig. 4a). The OD_572_/OD_600_ ratios (Fig. 4a) were consistent with the visually observed color changes (Fig. 4b).

We further investigated various other parameters of the assay, such as the incubation time between the addition of the indicator cells and the test sample, and the incubation time with resazurin before determination of the OD_572_/OD_600_ ratio (data not shown). The standard assay conditions were finally established as follows. The indicator cells were cultivated in the presence of the test samples for 24 h at 30 °C, and then incubated with resazurin for 4 h before assaying the OD_572_/OD_600_ ratio.

### Evaluation of the yeast-based REMA system

To evaluate the yeast-based assay conditions, the dependency of the color reaction on the dose of AZA was examined using serial dilutions of AZA ranging from 0.02 to 25 µM in the SG + Ade + Leu + His medium containing the assay cells at 1 × 10^5^ cells mL^−1^. The plates were incubated under the low- and high-CO_2_ conditions to determine the inhibitory effect on the hCAII and the cytotoxicity of AZA, respectively. We found that AZA significantly inhibited hCAII at a dose of 0.31 µM but no cytotoxicity was detected even at the highest concentration of AZA tested (25 µM) under the high CO_2_-condition (Fig. 5a, b). The results determined by the OD_572_/OD_600_ ratio (Fig. 5b) were consistent with the visual color-changes observed (Fig. 5c).

Comparison of the cell metabolism by the REMA method with that determined by the OD_660_ values revealed that the OD_660_ values showed consistent results with the OD_572_/OD_600_ ratio (Fig. 5a, b), and so could be used as a surrogate indicator of cell growth.

To further evaluate the specificity of the assay system, we compared the effect of AZA with those of avicennin, a CA isozyme IX inhibitor (Davis et al. 2013) and a FK506, a calcineurin inhibitor (Breuder et al. 1994; Liu et al. 1991; Rusnak and Mertz 2000; Shitamukai et al. 2000; Sugiura et al. 2001). The results showed that avicennin at 500 µM (its maximum solubility) showed only a weak inhibitory effect and FK506 up to 10 µM (its maximum solubility) showed no detectable inhibitory activity (Fig. 5c), indicating the high specificity of our assay system.

## Discussion

In this study, a novel yeast-based screening system for the detection of compounds that could inhibit the function of hCAII in vivo was successfully established using a high drug sensitive *nce103* null yeast mutant expressing hCAII as indicator cells. *S. cerevisiae* is highly tolerant of various drugs, due to the presence of ATP-binding cassette (ABC) transporter genes resulting in the poor permeability of these drugs through the yeast cell surface (Piecuch and Oblak 2014). Therefore, in this study the yeast strain BY25929 that has been disrupted for the genes encoding transcription activation factors of the ABC transporter genes (*PDR1, PDR3* and *YRR1*) and the ABC gene *YOR1* (Miyamoto et al. 2002), was further modified to attenuate the general permeability barriers for drugs by disruption of the *ERG3* gene, which encodes the C-6 desaturase of the ergosterol biosynthesis pathway (Hemmi et al. 1995). Then, the *NCE103* gene was further deleted along with the drug resistance genes to obtain a higher drug sensitive *nce103* null yeast strain designated as the AS03 strain. The Δ*nce103* strain deprived of its own CA can grow under a high (5%)—but not a low (ambient)-CO_2_ condition due to the difference in the availability of bicarbonate ions that are essential for cellular anaplerotic reactions (Aguilera et al. 2005). However, its growth defect phenotype when cultivated under a low-CO_2_ condition could be restored by overexpression of hCAII (Clark et al. 2004), or by only a low expression level of hCAII (Fig. 2).

The *GAL1* promoter was used to control the expression level of hCAII in the yeast transformants. *GAL1* is a strong-inducible promoter that strongly expresses when galactose, but not glucose, is present in the medium. The promoter contains the UAS_GAL_ 17-mer sites CGG-N_11_-CCG (Giniger et al. 1985) in four domains (Gal4 binding sites) that are recognized by the Gal4p homodimer transcription activator (Hong et al. 2008; Marmorstein et al. 1992). Cottier et al. (2006) reported that modifications in the number and type of Gal4 binding sites modulates the level of transcription of the HCMV protease gene, with 100%, 71%, 46% and 16% protein production levels, relative to the original *GAL1* promoter, with four, three, two and one Gal4 binding sites, respectively. In agreement with the above observation, our Western blot analysis and qRT-PCR results showed differential expression of hCAII in direct proportion with the number of Gal4 binding sites in the *GAL1* promoter of the expression construct as 100%, 82%, 62% and 39% for four, three, two and one Gal4 binding site(s), respectively (Fig. 1).

Taken together, the results of Figs. 1, 2 (at 0 µM AZA) and 3 (at 0 µM AZA) revealed that even the lowest level of hCAII (expressed from the promoter containing a single Gal4-binding site) was sufficient to complement the growth defect of the *nce103* null mutant under the low-CO_2_ condition.

**Fig. 3:**
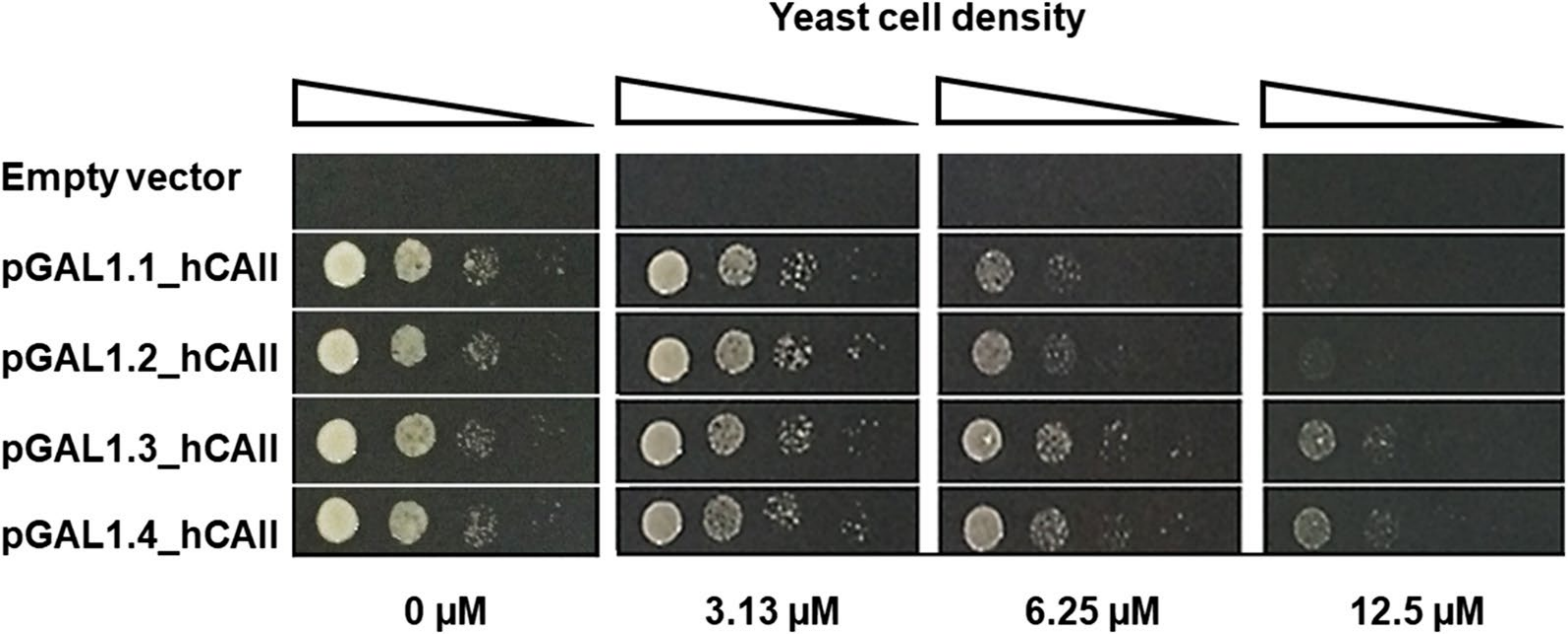
Drug (AZA) sensitivity of the AS03 strain expressing varying levels of hCAII. Tenfold dilutions of cell suspension of the yeast strain AS03 carrying either the empty pRS414 vector or one of pGAL1.1_hCAII, pGAL1.2_hCAII, pGAL1.3_hCAII or pGAL1.4_hCAII at concentrations of 10^6^, 10^5^, 10^4^ and 10^3^ cells mL^−1^ were spotted onto the surface of SG + Ade + Leu + His agar plates containing 0, 3.13, 6.25 or 12.5 μM of AZA and incubated at 30 °C for 3–4 days under a low CO_2_ condition. Images shown are representative from three independent repeats

Thus, using this transformant with the lowest expression level of hCAII (pGAL1.1_hCAII), we compared the sensitivity to AZA with three different yeast strains with deletions of various genes responsible for the general drug resistance of the cells under the low-CO_2_ condition. The spot test assay for AZA sensitivity of the three different strains of *nce103* null mutant background (AS01, AS02 and AS03; Table 1) revealed the strain showing the highest sensitivity to AZA was AS03(pGAL1.1_hCAII), followed by AS02(pGAL1.1_hCAII) and AS01(pGAL1.1_hCAII), respectively (Fig. 2). These results confirmed the high AZA sensitivity of the chosen indicator cells for our yeast-based assay and showed that CAIs that are effective against hCAII can be assayed for by monitoring the growth of these yeast strains (AS03, AS02 and AS01) relying on hCAII under the low-CO_2_ condition.

We chose the AS03 strain, as the most drug-sensitive strain, to examine the effect of the expressed levels of hCAII on the sensitivity to AZA. As expected, the degree of AZA resistance was positively correlated with the hCAII expression level (Fig. 3). Based on these data, the AS03(pGAL1.1_hCAII) transformant, which expressed the lowest level of hCAII (Fig. 1) and was the most sensitive strain, was selected as the indicator cells in the yeast-based inhibitor assay.

For the operational convenience of a HTS assay for a large number of samples using this yeast-based in vivo assay, we chose to examine the viability of the assay cells in liquid culture with resazurin as an indicator dye using a 96 well-plate format.

The principle of this yeast-based assay was that if tested compounds inhibit hCAII activity, then the *nce103* null mutant indicator cells would not be able to grow under the low CO_2_-condition but would grow under the high-CO_2_ condition if they were otherwise not cytotoxic to the yeast. Although the OD_660_ measurement provided an easy and efficient way to quantify yeast growth over time, metabolic indicators of the cell density, based on the metabolism-dependent reduction of colorimetric dyes, provide an alternative measurement of yeast cell density as well as providing an indicator of yeast vitality (Goughenour et al. 2015). Here, we used the colorimetric indicator resazurin that can also be used as a more objective qualitative indicator. Although the use of resazurin as indicator dye for cell viability has several advantages, care must be taken in setting up the REMA to get a correlation between the OD_572_/OD_600_ ratio and cell turbidity (OD_660_).

During the resazurin incubation, the color of the solution containing the indicator cells in the absence of AZA (negative control) changed from blue to pink and this color change was determined by measuring the OD_572_ value for resorufin and OD_600_ for resazurin and expressed as the OD_572_/OD_600_ ratio. In the absence of AZA, the cells were able to grow under the low-CO_2_ condition relying on their expressed hCAII, which led to the color change, while the sample without the indicator cells remained blue. In the sample containing the indicator cells and AZA at a concentration sufficient to inhibit the hCAII, the color remained blue with an OD_572_/OD_600_ ratio that was lower than that without the inhibitor (negative control).

**Fig. 4:**
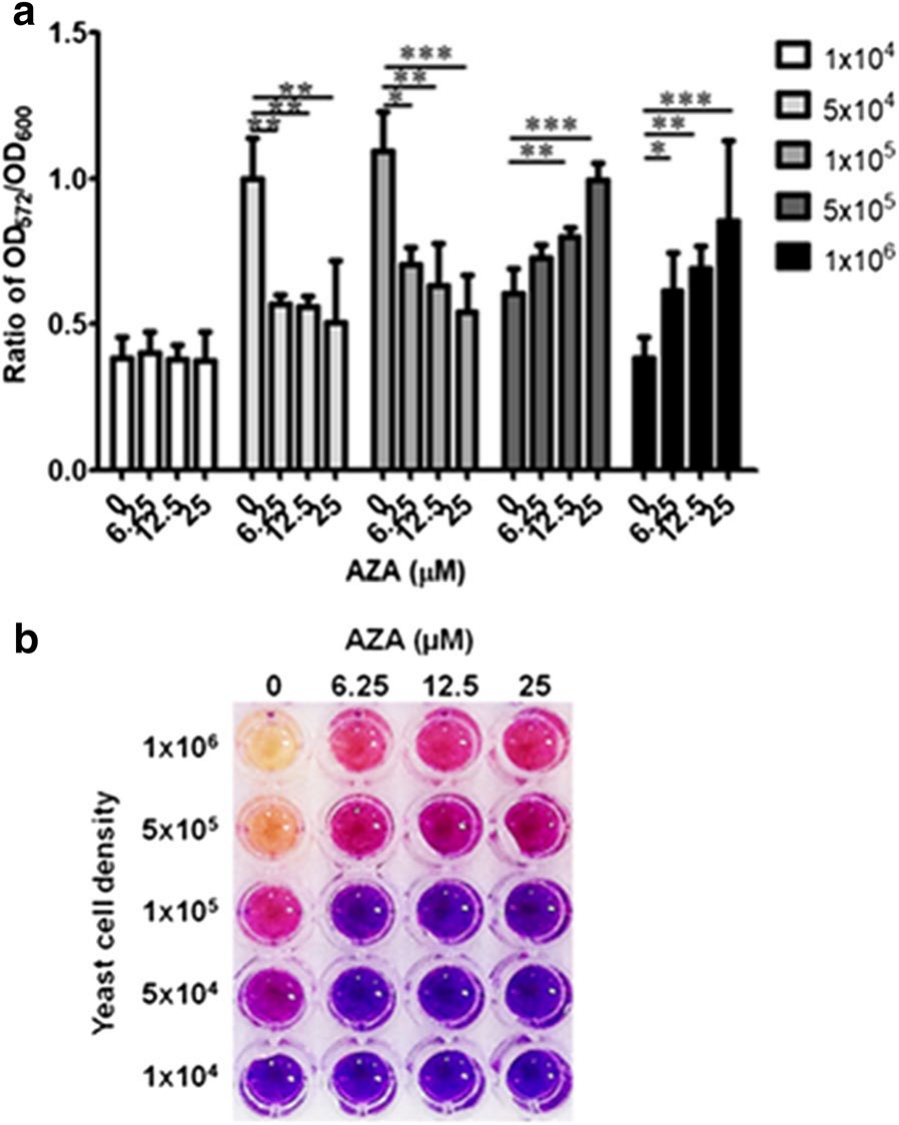
Optimization of the initial cell concentration using the REMA. A suspension of the yeast strain AS03(pGAL1.1_hCAII) at varying final concentrations of 1 × 10^6^, 5 × 10^5^, 1 × 10^5^, 5 × 10^4^ and 1 × 10^4^ cells mL^−1^ in 100 µL of SG + Ade + Leu + His medium were added to each well of a 96-well plate with varying concentrations of AZA (0, 6.25, 12.5 and 25 µM). The plates were incubated at 30 °C for 24 h whereupon resazurin was added and 4 h later the OD_572_ and OD_600_ values were measured and expressed as **a** the OD_572_/OD_600_ ratio and **b** visual observation. Data are shown as **a** the mean ± 1 SD derived from three independent repeats, where *, ** and *** represent a significant difference at p < 0.05, p < 0.01 and p < 0.001 levels, respectively, compared to that of the untreated control group, and **b** representative image of three independent repeats

An optimal initial yeast cell density is one of the important parameters of the assay. With too high cell density (0.5–1 × 10^6^ cells mL^−1^), the OD_572_/OD_600_ ratio in the test-wells containing AZA was higher than that in the negative control (Fig. 4a), which is due to the extensive reduction of resazurin into resorufin and then hydroresorufin (colorless) in the control-wells (Ramsdell et al. 1935). Whereas, an over twofold difference in the OD_572_/OD_600_ ratio between the control and AZA-treated cells was noted when using a yeast cell density of 0.5–1 × 10^5^ cells mL^−1^ (Fig. 4a). This observation is consistent with those determined by visualization of the color change (Fig. 4b). However, at a lower yeast cell density (1 × 10^4^ cells mL^−1^) there was no significant difference in the OD_572_/OD_600_ ratio between the control and AZA-treated wells (Fig. 4a). Therefore, under these conditions 0.5–1 × 10^5^ cells mL^−1^ was the appropriate initial yeast cell density.

**Fig. 5:**
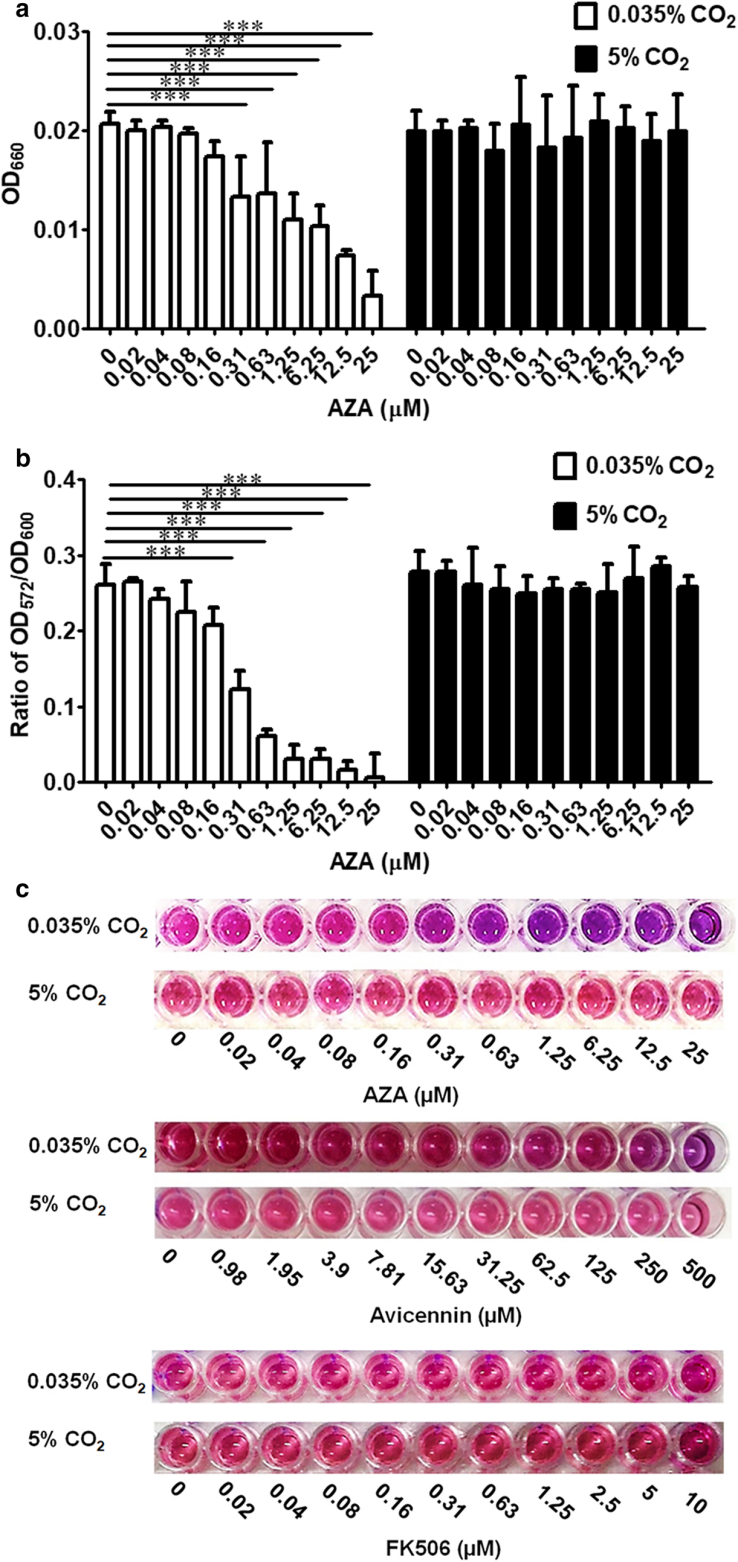
Determination of the minimal effective dose of the tested compounds. Serial dilutions of either AZA (0–25 µM), avicennin (0–500 µM) or FK506 (0–10 µM) were added in wells of a 96-well plate containing 0.5 × 10^5^ cells mL^−1^ of AS03(pGAL1.1_hCAII) cells and incubated at 30 °C under a high- or low-CO_2_ condition. **a** The growth of the yeast strain treated with AZA was determined by the OD_660_ value prior to adding resazurin into each well. **b** Then, after 4 h, the OD_572_ and OD_600_ values were measured and expressed as the OD_572_/OD_600_ ratio. **c** The colorimetric results of the assay for three tested compounds were also observed visually. Data are shown as (**a**, **b)** the mean ± 1 SD derived from three independent repeats, where *, ** and *** represent a significant difference at p < 0.05, p < 0.01 and p < 0.001 levels, respectively, compared to that of the untreated control group, and **c** a representative image of three independent repeats

The result showed that the minimal effective dose of AZA determined by the optimized REMA method was 0.31 µM (Fig. 5), which was 20- to 40-fold lower than that determined from the spot test on agar plates (6.25–12.5 µM; Fig. 3). Therefore, the REMA method significantly increased the sensitivity for the detection of this inhibitor compared to the spot test on agar plate. Furthermore, this assay system showed a potentially high specificity (Fig. 5c).

In this study, an in vivo screening method for CAIs that inhibit hCAII was developed utilizing a drug sensitive *nce103* null mutant strain expressing and relying on a low level of hCAII as indicator cells in combination with a REMA assay for the HTS of potential novel drugs. The yeast-based in vivo assay system has several advantages over the conventional in vitro biochemical screening assay as follows. Since this screening procedure is based on living yeast cells, in contrast to the conventional in vitro biochemical screening procedures, only compounds that permeate through the cell surface, and compounds that are metabolically stable in vivo can be screened.

Since the assay is based on the growth inhibition of the assay cells, cytotoxic compounds will be also selected by the screening. These compounds, however, can be easily eliminated by examining the growth of the assay cells under the high-CO_2_ condition, where the cells do not depend on the hCAII activity for growth.

This screening procedure will potentially facilitate a high throughput screening system for detection of safer hCAII inhibitors that could be further developed as a drug, especially for tropical use in glaucoma treatment. However, the candidates obtained from this screening procedure still require further investigation for their hCAII specificity by screening for inhibitory activity against other CA isozymes.

## Abbreviations

CA: carbonic anhydrase
hCAII: human carbonic anhydrase isozyme II
CAI: carbonic anhydrase inhibitor

## References

[CR1] Aguilera J, Van Dijken JP, De Winde JH, Pronk JT (2005). Carbonic anhydrase (Nce103p): an essential biosynthetic enzyme for growth of *Saccharomyces cerevisiae* at atmospheric carbon dioxide pressure. Biochem J.

[CR2] Amberg DC, Burke DJ, Burke D, Strathern JN (2005). Method in yeast genetics 2005 edition.

[CR3] Bilsland E, Sparkes A, Williams K, Moss HJ, de Clare M, Pir P, Rowland J, Aubrey W, Pateman R, Young M, Carrington M, King RD, Oliver SG (2013). Yeast-based automated high-throughput screens to identify anti-parasitic lead compounds. Open Biol.

[CR4] Breuder T, Hemenway CS, Movva NR, Cardenas ME, Heitman J (1994). Calcineurin is essential in cyclosporin A- and FK506-sensitive yeast strains. Proc Natl Acad Sci USA.

[CR5] Carta F, Supuran CT (2013). Diuretics with carbonic anhydrase inhibitory action: a patent and literature review (2005–2013). Expert Opin Ther Pat.

[CR6] Clark D, Rowlett RS, Coleman JR, Klessig DF (2004). Complementation of the yeast deletion mutant DeltaNCE103 by members of the beta class of carbonic anhydrases is dependent on carbonic anhydrase activity rather than on antioxidant activity. Biochem J.

[CR7] Cottier V, Barberis A, Luthi U (2006). Novel yeast cell-based assay to screen for inhibitors of human cytomegalovirus protease in a high-throughput format. Antimicrob Agents Chemother.

[CR8] Daniel A, Anthony A, Boris B, Michael F, Igor S (2005). Drug discovery using yeast as a model system: a functional genomic and proteomic view. Curr Proteomics.

[CR9] Davis RA, Vullo D, Maresca A, Supuran CT, Poulsen SA (2013). Natural product coumarins that inhibit human carbonic anhydrases. Bioorgan Med Chem.

[CR10] Del Prete S, Vullo D, Fisher GM, Andrews KT, Poulsen SA, Capasso C, Supuran CT (2014). Discovery of a new family of carbonic anhydrases in the malaria pathogen *Plasmodium falciparum*—the eta-carbonic anhydrases. Bioorg Med Chem Lett.

[CR11] Gietz RD, Schiestl RH, Willems AR, Woods RA (1995). Studies on the transformation of intact yeast-cells by the Liac/S-DNA/Peg procedure. Yeast.

[CR12] Giniger E, Varnum SM, Ptashne M (1985). Specific DNA binding of GAL4, a positive regulatory protein of yeast. Cell.

[CR13] Goughenour KD, Balada-Llasat JM, Rappleye CA (2015). Quantitative microplate-based growth assay for determination of antifungal susceptibility of *Histoplasma capsulatum* yeasts. J Clin Microbiol.

[CR14] Gueldener U, Heinisch J, Koehler GJ, Voss D, Hegemann JH (2002). A second set of loxP marker cassettes for Cre-mediated multiple gene knockouts in budding yeast. Nucleic Acids Res.

[CR15] Hemmi K, Julmanop C, Hirata D, Tsuchiya E, Takemoto JY, Miyakawa T (1995). The physiological roles of membrane ergosterol as revealed by the phenotypes of Syr1/Erg3 null mutant of *Saccharomyces*-*cerevisiae*. Biosci Biotech Biochem.

[CR16] Hong M, Fitzgerald MX, Harper S, Luo C, Speicher DW, Marmorstein R (2008). Structural basis for dimerization in DNA recognition by Gal4. Structure.

[CR17] Krungkrai J, Scozzafava A, Reungprapavut S, Krungkrai SR, Rattanajak R, Kamchonwongpaisan S, Supuran CT (2005). Carbonic anhydrase inhibitors. Inhibition of *Plasmodium falciparum* carbonic anhydrase with aromatic sulfonamides: towards antimalarials with a novel mechanism of action?. Bioorg Med Chem.

[CR18] Liang SD, Marmorstein R, Harrison SC, Ptashne M (1996). DNA sequence preferences of GAL4 and PPR1: how a subset of Zn2 Cys6 binuclear cluster proteins recognizes DNA. Mol Cell Biol.

[CR19] Lindskog S (1997). Structure and mechanism of carbonic anhydrase. Pharmacol Ther.

[CR20] Liu J, Farmer JD, Lane WS, Friedman J, Weissman I, Schreiber SL (1991). Calcineurin is a common target of cyclophilin–cyclosporin A and FKBP-FK506 complexes. Cell.

[CR21] Livak KJ, Schmittgen TD (2001). Analysis of relative gene expression data using real-time quantitative PCR and the 2(−Delta Delta C(T)) method. Methods.

[CR22] Lomelino CL, Supuran CT, McKenna R (2016). Non-classical inhibition of carbonic anhydrase. Int J Mol Sci.

[CR23] Marmorstein R, Carey M, Ptashne M, Harrison SC (1992). DNA recognition by GAL4: structure of a protein–DNA complex. Nature.

[CR24] Masini E, Carta F, Scozzafava A, Supuran CT (2013). Antiglaucoma carbonic anhydrase inhibitors: a patent review. Expert Opin Ther Pat.

[CR25] Miyamoto Y, Machida K, Mizunuma M, Emoto Y, Sato N, Miyahara K, Hirata D, Usui T, Takahashi H, Osada H, Miyakawa T (2002). Identification of *Saccharomyces cerevisiae* isoleucyl-tRNA synthetase as a target of the G(1)-specific inhibitor reveromycin A. J Biol Chem.

[CR26] Monti SM, Supuran CT, De Simone G (2013). Anticancer carbonic anhydrase inhibitors: a patent review (2008–2013). Expert Opin Ther Pat.

[CR27] Panthan B (2011) Expression of *Plasmodium falciparum* carbonic anhydrase in *Saccharomyces cerevisiae* deletion mutant ∆nce103. Master of Science (Microbiology), Chulalongkorn University

[CR28] Piecuch A, Oblak E (2014). Yeast ABC proteins involved in multidrug resistance. Cell Mol Biol Lett.

[CR29] Rampersad SN (2012). Multiple applications of Alamar Blue as an indicator of metabolic function and cellular health in cell viability bioassays. Sensors (Basel).

[CR30] Ramsdell GA, Johnson WMT, Evans FR (1935). Investigation of resazurin as an indicator of the sanitary condition of milk. J Dairy Sci.

[CR31] Rusnak F, Mertz P (2000). Calcineurin: form and function. Physiol Rev.

[CR32] Sambrook J, Fritsch EF, Maniatis T (1989). Molecular cloning: a laboratory manual.

[CR33] Scozzafava A, Supuran CT, Carta F (2013). Antiobesity carbonic anhydrase inhibitors: a literature and patent review. Expert Opin Ther Pat.

[CR34] Shitamukai A, Mizunuma M, Hirata D, Takahashi H, Miyakawa T (2000). A positive screening for drugs that specifically inhibit the Ca^2+^-signaling activity on the basis of the growth promoting effect on a yeast mutant with a peculiar phenotype. Biosci Biotechnol Biochem.

[CR35] Sly WS, Hu PY (1995). Human carbonic anhydrases and carbonic anhydrase deficiencies. Annu Rev Biochem.

[CR36] Sugiura R, Sio SO, Shuntoh H, Kuno T (2001). Molecular genetic analysis of the calcineurin signaling pathways. Cell Mol Life Sci.

[CR37] Supuran CT (2008). Carbonic anhydrases: novel therapeutic applications for inhibitors and activators. Nat Rev Drug Discov.

[CR38] Supuran CT, Scozzafava A (2000). Carbonic anhydrase inhibitors and their therapeutic potential. Expert Opin Ther Pat.

